# Synthesis of SiC/Ag/Cellulose Nanocomposite and Its Antibacterial Activity by Reactive Oxygen Species Generation

**DOI:** 10.3390/nano6090171

**Published:** 2016-09-13

**Authors:** Andrzej Borkowski, Tomasz Cłapa, Mateusz Szala, Arkadiusz Gąsiński, Marek Selwet

**Affiliations:** 1 Faculty of Geology, University of Warsaw, Żwirki i Wigury 93, 02-089 Warsaw, Poland; agasin@uw.edu.pl; 2 Department of General and Environmental Microbiology, Poznań University of Life Sciences, ul. Szydłowska 50, 60-656 Poznań, Poland; tom.clapa@gmail.com (T.C.); marek.selwet@gmail.com (M.S.); 3 Faculty of Advanced Technologies and Chemistry, Military University of Technology, Kaliskiego 2, 00-908 Warsaw, Poland; mateusz.szala@wat.edu.pl

**Keywords:** Ag nanoparticles, antibacterial activity, cellulose, reactive oxygen species, silicon carbide nanocomposite

## Abstract

We describe the synthesis of nanocomposites, based on nanofibers of silicon carbide, silver nanoparticles, and cellulose. Silver nanoparticle synthesis was achieved with chemical reduction using hydrazine by adding two different surfactants to obtain a nanocomposite with silver nanoparticles of different diameters. Determination of antibacterial activity was based on respiration tests. Enzymatic analysis indicates oxidative stress, and viability testing was conducted using an epifluorescence microscope. Strong bactericidal activity of nanocomposites was found against bacteria *Escherichia coli* and *Bacillus cereus*, which were used in the study as typical Gram-negative and Gram-positive bacteria, respectively. It is assumed that reactive oxygen species generation was responsible for the observed antibacterial effect of the investigated materials. Due to the properties of silicon carbide nanofiber, the obtained nanocomposite may have potential use in technology related to water and air purification. Cellulose addition prevented silver nanoparticle release and probably enhanced bacterial adsorption onto aggregates of the nanocomposite material.

## 1. Introduction

Following a period of intensive fundamental research on nanostructured materials, their possible use in a variety of technologies is a new direction of research. Opportunities exist in the application of nanostructured materials in medicine, exploring bactericidal and cytotoxic properties, and in technologies related to biotechnology and the chemical industry. An important potential area of application of nanostructured materials is in water- and air-treatment technologies. For this reason, many studies have been published, where the possibility of synthesis of a wide variety of nanocomposites, based primarily on single- and multi-walled carbon nanotubes, and graphene, combined with metal and metal oxide nanoparticles, have been presented [1,2,3,4]. The most interesting studies related to nanocomposites include those where silver, zinc oxide, and titanium oxide nanoparticles were used. Very often, nanostructured carbon is treated as a nanoparticle carrier. One such carrier is silicon carbide (SiC) nanofibers, which are chemically more stable than nanostructured carbon, and, therefore, have potential application in technologies based on filtering methods.

SiC nanostructures are comprised mainly of SiC nanofibers and nanorods, which may be obtained using combustion synthesis [5,6]. In this method, after product purification, a material can be produced that consists almost exclusively of nanostructured SiC. Nanofibers, nanorods, and SiC nanoparticles can exhibit antibacterial properties by injury of cell membrane integrity and generation of oxidative stress [7,8,9,10]. Such a bactericidal mechanism has been observed with various nanostructured materials [11,12]. Of particular interest are metal nanoparticles, mainly silver nanoparticles (AgNPs), which have strong bactericidal properties [13,14,15,16,17,18]. The main mechanisms of the bactericidal action of AgNPs are: (1) interaction of AgNPs with the bacterial membranes; (2) the generation of reactive oxygen species (ROS), which may lead to a number of adverse processes in cells, such as changes in enzyme activity, lipid peroxidation, growth inhibition, and cell death; and (3) release of toxic Ag ions. It is possible that the release of Ag ions is the most important factor leading to cell injury due to contact with AgNPs. Kittler et al. [19] concluded that the toxicity of AgNPs increased during storage due to the dissociation of Ag ions.

The use of AgNPs and other metal and metal oxide nanoparticles may be limited to the synthesis of a suspension in a suitable liquid or semi-liquid medium, where the suspension should have the desired properties that limit microbial growth. For solids, such an approach may be more cumbersome; therefore, a variety of nanocomposite materials containing metal nanoparticles may be required. For instance, the bactericidal activity of nanocomposites based on graphene oxide/chitosan/ZnO nanoparticles has been demonstrated [4]. The most common AgNPs or metal nanoparticles synthesis method is chemical reduction under different conditions using, for example, monosaccharides [14] or hydrazine [20]. The addition of surfactants during the reduction allows for control of nanoparticle size and aggregate formation.

Cellulose has often been used as a material for nanocomposites with silver. It can be concluded that cellulose-based nanocomposites could be implemented as a promising antibacterial material, especially for food packaging and transport [21,22]. Dallas et al. [23] have reported that the cellulose-based nanocomposites and their derivatives are gaining importance because of their implementation in science and technology. They pay attention to possible applications, such as high-performance composite materials, organoclay-exfoliated cellulose with improved mechanical properties, or nanocomposites for biomedical applications. An interesting application of cellulose-based nanocomposites is the production of bacterial cellulose impregnated by AgNPs for use as a wound dressing [24].

The aim of this study was to use SiC nanofibers as a carrier for AgNPs. We present the synthesis of a nanocomposite material based on SiC nanofibers, AgNPs, and cellulose (CE). CE was added to stabilize the system and to prevent the release of AgNPs. The obtained nanocomposites were used in microbiological tests to determine their potential bactericidal properties. AgNPs were synthesized under two different surfactant addition conditions to obtain nanocomposites with various-sized AgNPs, and to evaluate the impact of the nanoparticles on the bactericidal activity of the nanocomposites.

## 2. Results and Discussion

### 2.1. Nanocomposite Characterization

**Figure 1:**
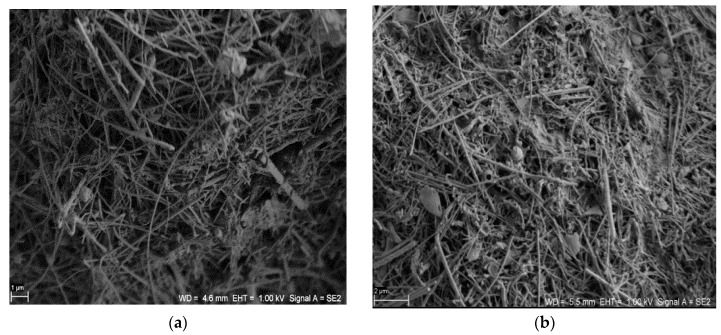
Scanning electron microscopy (SEM) images of (**a**) silicon carbide (SiC) nanofibers (Scale bar: 1 μm) and (**b**) the SiC/cellulose (CE) nanocomposite. (Scale bar: 2 μm).

**Figure 2:**
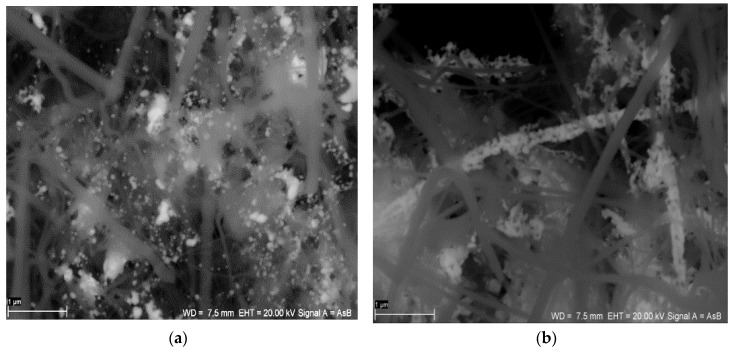
SEM images of (**a**) the SiC/Ag/CE/SDS nanocomposite (AgNPs synthesized via SDS–sodium dodecyl sulphate) and (**b**) the SiC/Ag/CE/T nanocomposite (AgNPs synthesized via Tween). (Scale bar: 1 μm).

The structures of the SiC nanofibers and nanocomposite SiC/CE are presented in Figure 1. There were no significant microscopic differences between the SiC nanofibers and the SiC/CE nanocomposite; only a portion of the fibers appeared to be joined closely together and were partially covered by CE. Based on weight analysis and the composition of the reagent used, the content of cellulose in the nanocomposite was about 7%–9% (*w*/*w*). The scanning electron microscopy (SEM) images of SiC/Ag/CE/SDS (AgNPs synthesized via SDS–sodium dodecyl sulphate) and SiC/Ag/CE/T nanocomposites (AgNPs synthesized via Tween) are shown in Figure 2. Micrographs were taken in backscattered electron mode to better visualize the silver nanoparticles.

**Figure 3:**
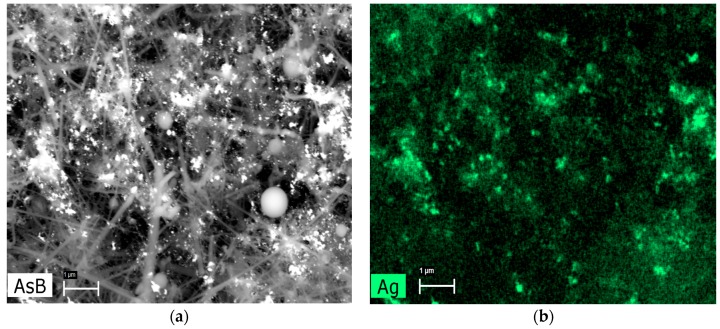
Example of energy-dispersive X-ray spectroscopy mapping. (**a**) SEM image in backscattered electron mode and (**b**) mapping indicating the distribution of Ag in the nanocomposite SiC/Ag/CE/SDS. AsB: backscattered electron mode. (Scale bar: 1 μm).

AgNPs distribution was shown by energy-dispersive X-ray spectroscopy (Figure 3). For the first nanocomposite, AgNPs were more scattered, and for SiC/Ag/CE/T, AgNPs were associated strongly with the SiC nanofibers. It is possible that the linkage between the SiC nanofibers and AgNPs is a physical phenomenon rather than a chemical interaction. Therefore, it is also possible that AgNPs could be released from the nanocomposites. To minimize this effect, the obtained nanocomposite suspension was centrifuged and washed in deionized water until the unbound AgNPs were washed out. After such treatment, there was no peak at 390–420 nm in the filtrate. In preliminary experiments, it was found that CE prevented the release of AgNPs. Synthesis of the nanocomposite without the CE resulted in a more extensive release of AgNPs, which was observed as a peak at 390–420 nm after washing of the obtained nanocomposites. Thus, the addition of CE prevented the dispersion of non-associated AgNPs with SiC nanofibers in aqueous suspension. Furthermore, during the synthesis of SiC/Ag/CE, the addition of CE resulted in the binding of remaining AgNPs.

**Figure 4:**
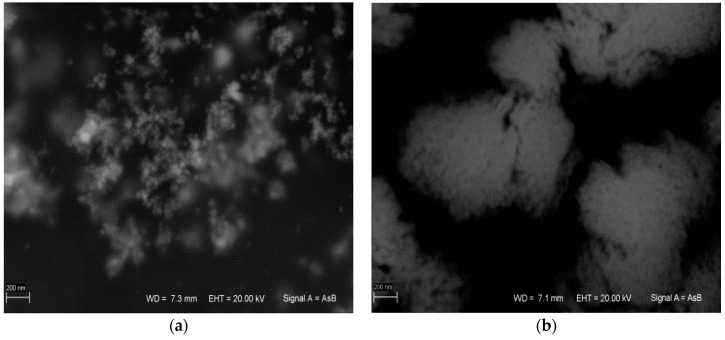
(**a**) Ag nanoparticles synthesized via SDS (AgNP/SDS); and (**b**) Ag nanoparticles synthesized via Tween 20 (AgNP/T). (Scale bar: 200 nm).

**Figure 5:**
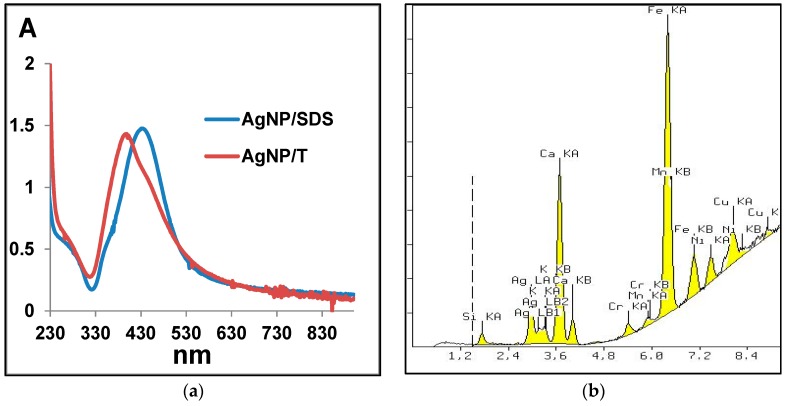
(**a**) Ultraviolet-visible (UV-VIS) spectra of Ag nanoparticles; and (**b**) an example of X-ray fluorescence spectrometry (XRF) spectrum of the nanocomposite SiC/Ag/CE/SDS. A: Absorbance.

AgNPs synthesis was performed in 1% SDS or Tween 20 solution. Surfactant addition mainly prevents the formation of the so-called silver mirror (Tollens reaction). SEM analysis of the obtained AgNPs revealed that the AgNP/SDS particle size ranged from 15 to 50 nm, whereas for AgNP/T, particles formed aggregates larger than 200 nm. These aggregates consisted of ~10-nm diameter nanoparticles (Figure 4). The difference between the obtained AgNPs was also visible in the ultraviolet-visible (UV-VIS) spectrum (Figure 5a).

**Figure 6:**
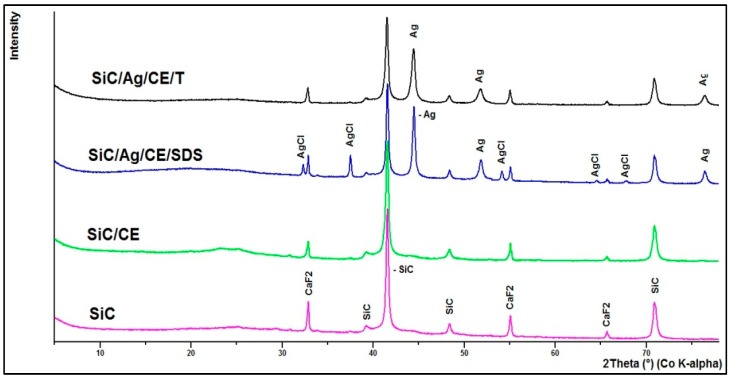
X-ray powder diffraction (XRD) of investigated materials.

Based on X-ray fluorescence spectrometry (XRF) analysis, it can be stated that the silver content in the nanocomposites was 2.88% and 2.33% for SiC/Ag/CE/SDS and SiC/Ag/CE/T, respectively. The XRF spectrum (Figure 5b) revealed the presence of elements contained in the tested nanomaterial, namely Ag and Si, and elements derived from various contaminants, such as Ca from substrates for combustion synthesis. The Fe signal was most likely derived from the device casing. These contaminants were comparable in the studied materials. X-ray powder diffraction (XRD) analysis (Figure 6) revealed characteristic peaks for SiC and Ag for SiC/Ag/CE nanocomposites. Calcium fluoride (CaF_2_) was a contaminant from combustion synthesis.

### 2.2. Respirometric Analysis

**Figure 7:**
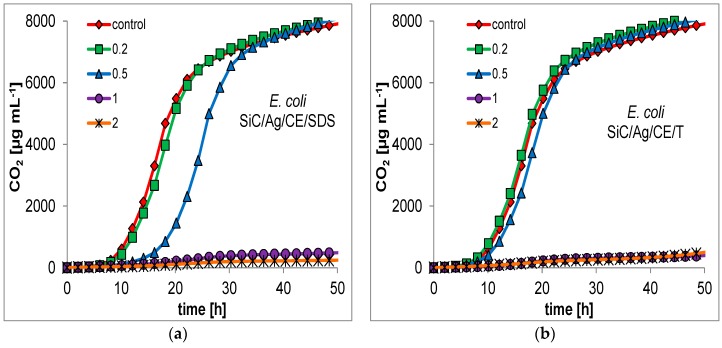
Respirometric curves showing the growth of *E. coli* at different concentrations (mg·mL^−1^; as shown in the insets) of (**a**) SiC/Ag/CE/SDS; (**b**) SiC/Ag/CE/T.

**Figure 8:**
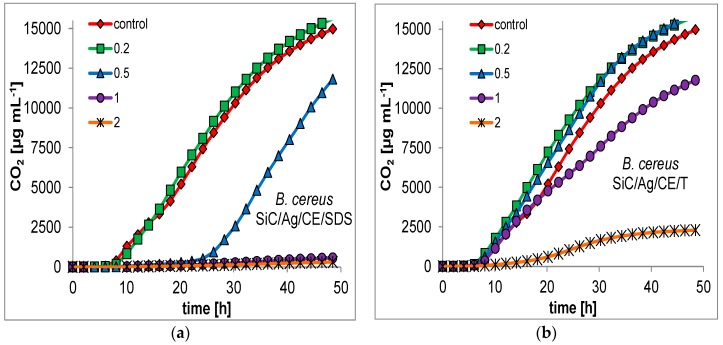
Examples of respirometric curves of growth of *B. cereus* at different concentration (legend, mg·mL^−1^) of nanocomposites. (**a**) SiC/Ag/CE/SDS; (**b**) SiC/Ag/CE/T.

For many nanoparticles, the minimum inhibitory concentration (MIC) is usually measured as a primary indication of bactericidal properties. This determination can be performed by evaluation of the optical density in microbial cultures, or on microtiter plates with a growth indicator [18,25]. This approach may be unreliable for the presented nanocomposites. SiC nanofibers form quite heterogeneous mixtures in an aqueous suspension, which makes it difficult to conduct a series dilution of the test material. Therefore, it was decided to evaluate the bactericidal properties based on respirometric curves, which determine the CO_2_ production in small batch cultures, with the addition of the studied nanomaterials (Figure 7 and Figure 8). In addition, the curves allowed for a better comparison of the bacterial response to the presence of toxic substances. Based on the respirometric analysis, SiC nanofibers and the SiC/CE nanocomposite did not significantly inhibit microbial growth in the range 0.2–2 mg·mL^−1^ (curves not shown; their course was very similar to the control). However, for SiC/Ag/CE/SDS and SiC/Ag/CE/T, there were significant differences in the rate of CO_2_ production. For *E. coli*, the MICs for SiC/Ag/CE/SDS and SiC/Ag/CE/T were 2 mg·mL^−1^ and slightly above 2 mg·mL^−1^, respectively. MIC values for *B. cereus* were similar, but it appeared that *B. cereus* had a higher sensitivity to SiC/Ag/CE/SDS and a lower sensitivity for SiC/Ag/CE/T relative to *E. coli*.

### 2.3. Catalase and Dehydrogenase Activities

**Figure 9:**
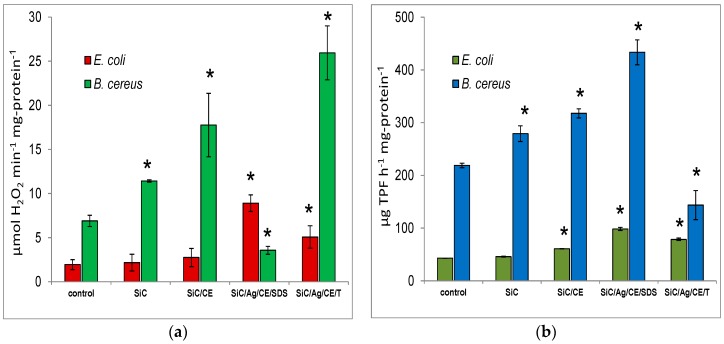
(**a**) Catalase activity and (**b**) dehydrogenase activity after treatment of *E. coli* or *B. cereus* with SiC nanofibers and nanocomposites at a concentration of 1 mg·mL^−1^. Significant differences (*) compared to controls and the standard deviation are also indicated.

Many studies indicate that the two most important factors that affect nanostructured material toxicity are mechanical injury of the cell membrane integrity, and reactive oxygen species (ROS) generation and oxidative stress. The loss of cell membrane integrity and ROS generation may also lead to lipid peroxidation [26,27]. In this study, we measured the impact of SiC/Ag nanocomposites on microbial growth and CO_2_ production. Significant catalase and dehydrogenase activities were observed in treated cultures (Figure 9a). Catalase activity is responsible for the decomposition of hydrogen peroxide, which can be generated in cells as a result of dismutation of the superoxide radical (O_2_^−^) catalyzed by superoxide dismutase. It can be assumed, therefore, that the presence of ROS may lead to increased activity of an important enzyme that protects against ROS. A significant increase in catalase activity was observed in *Escherichia coli* cultures in the presence of SiC/Ag/CE/SDS and SiC/Ag/CE/T nanocomposites. For *Bacillus cereus*, growth was observed in all cultures with nanomaterials except for SiC/Ag/CE/SDS. This was probably because of insufficient bacterial growth within a given time, and an apparent reduction in enzymatic activity was observed. This experiment indicated the greater sensitivity of *B. cereus* to SiC/Ag/CE/SDS compared to the other nanomaterials. Chowdhuri et al. [4] showed a similar decrease in catalase activity in cultures of *E. coli* and *Staphylococcus aureus* in the presence of a nanocomposite based on graphene, chitosan, and ZnO nanoparticles. However, it can be assumed that, in this case, results were also affected by inadequate microbial growth. The increase in catalase activity was also demonstrated in cultures with SiC/CE, particularly for *B. cereus*. It is possible that CE increased the adhesion of negatively charged bacterial cells onto SiC/CE aggregates formed in an aqueous suspension. A similar effect was observed by Chowdhuri et al. [4] for graphene oxide and a chitosan nanocomposite.

Many studies indicate that the two most important factors that affect nanostructured material toxicity are mechanical injury of the cell membrane integrity, and reactive oxygen species (ROS) generation and oxidative stress. The loss of cell membrane integrity and ROS generation may also lead to lipid peroxidation [26,27]. In this study, we measured the impact of SiC/Ag nanocomposites on microbial growth and CO_2_ production. Significant catalase and dehydrogenase activities were observed in treated cultures (Figure 9a). Catalase activity is responsible for the decomposition of hydrogen peroxide, which can be generated in cells as a result of dismutation of the superoxide radical (O_2_^−^) catalyzed by superoxide dismutase. It can be assumed, therefore, that the presence of ROS may lead to increased activity of an important enzyme that protects against ROS. A significant increase in catalase activity was observed in *Escherichia coli* cultures in the presence of SiC/Ag/CE/SDS and SiC/Ag/CE/T nanocomposites. For *Bacillus cereus*, growth was observed in all cultures with nanomaterials except for SiC/Ag/CE/SDS. This was probably because of insufficient bacterial growth within a given time, and an apparent reduction in enzymatic activity was observed. This experiment indicated the greater sensitivity of *B. cereus* to SiC/Ag/CE/SDS compared to the other nanomaterials. Chowdhuri et al. [4] showed a similar decrease in catalase activity in cultures of *E. coli* and *Staphylococcus aureus* in the presence of a nanocomposite based on graphene, chitosan, and ZnO nanoparticles. However, it can be assumed that, in this case, results were also affected by inadequate microbial growth. The increase in catalase activity was also demonstrated in cultures with SiC/CE, particularly for *B. cereus*. It is possible that CE increased the adhesion of negatively charged bacterial cells onto SiC/CE aggregates formed in an aqueous suspension. A similar effect was observed by Chowdhuri et al. [4] for graphene oxide and a chitosan nanocomposite.

The dehydrogenase activity in the same treated cultures is presented in Figure 9b. The activity increased for *E. coli* in the presence of SiC/CE, SiC/Ag/CE/SDS, and SiC/Ag/CE/T. The greatest activity was found for *E. coli* and *B. cereus* in cultures with SiC/Ag/CE/SDS. Surprisingly, however, the dehydrogenase activity in *B. cereus* cultures with SiC/Ag/CE/T decreased. It seems that, similar to the case of catalase, the observed effect may be brought about by differences in the growth kinetics or other factors that are not obvious. The addition of tetrazolium substrates to the cell culture leads to their reduction, and the formation of a colored formazan, mainly because of dehydrogenase activity. However, under certain conditions, these substrates may also be reduced, incidentally, as a result of free radical reactions with the superoxide radical [28]. Regardless of the main reaction responsible for the reduction of tetrazolium substrates, it can be assumed that the measured dehydrogenase activity is a reflection of the physiological state of the cells. Dehydrogenase activity, oxidative stress, ROS generation, and processes leading to the uncoupling of phosphorylation and energy dissipation take place in these cells.

### 2.4. Viability Test

**Figure 10:**
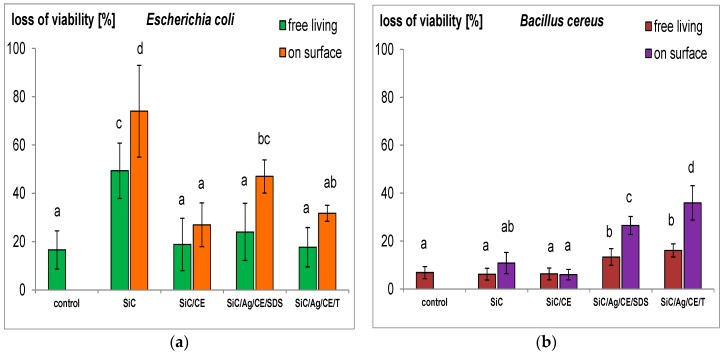
Loss of viability (%) of free-living cells and those adsorbed onto the surface of aggregates of nanofibers SiC and nanocomposites. (**a**) *Escherichia coli*; and (**b**) *Bacillus cereus*. The same letters indicate that the values do not differ significantly at *p* < 0.05. The error bars indicate the standard deviation.

One of the important effects of the bacteria–nanostructure interaction is the loss of cell membrane integrity [11,29], which may increase the permeation of dyes, such as propidium iodide (PI), into cells. In a normal physiological state, such dyes are removed from cells. Contact with the nanostructures may lead to mechanical damage, as was often demonstrated in the case of single- and multi-walled carbon nanotubes [11,30], or on SiC in the form of nanofibers and nanorods [9,10]. In this study, an interesting effect was noted. In *E. coli* cultures treated with SiC nanofibers, a loss of viability was observed, especially in bacteria adsorbed on the surface of the SiC nanofiber aggregates (Figure 10). This result is consistent with previously published data [9]. However, for SiC/CE, this effect disappeared, and the viability was similar to that of the control. The CE in the SiC/CE most likely reduced the mechanical interactions between bacterial cells and SiC nanofibers. A loss of cell viability was also observed in bacteria treated using nanocomposites with silver. However, this effect may be caused, more by the properties of AgNPs, than by injury to cell membranes caused by SiC. This hypothesis can be confirmed by results from cultures of *B. cereus*. The loss of viability was significantly greater than the control only for SiC/Ag/CE/SDS and SiC/Ag/CE/T. This effect was not observed in the other cases, probably because of the structure of the Gram-positive bacterial cell wall [31].

## 3. Materials and Methods

### 3.1. Synthesis of SiC Nanofibers

SiC was prepared by combustion [6]. The combustion mixture was prepared by dry mixing calcium disilicide (CaSi_2_) and poly (tetrafluoroethene) powders in a ceramic mortar. After pressing the powders into a cylindrical pellet, 5 g of sample were placed in a graphite crucible in a stainless steel autoclave (350 mL), which was subsequently filled with helium at an initial pressure of 1.0 MPa. Combustion was initiated with an electrically-heated resistance wire (0.1-mm diameter). Spongy combustion products were removed from the autoclave with water. The suspension was filtered, and the gray deposit obtained was purified in a three-step process: heating in 98% H_2_SO_4_, calcination in air (700 °C), and heating in 25% NaOH, and washing with plenty of water. Nanomaterials were characterized using X-ray powder diffraction (XRD), Raman spectroscopy, elemental analysis, scanning electron microscopy (SEM), and transmission electron microscopy [5]. The SiC nanofibers were covered with a thin layer of silica.

### 3.2. Synthesis of SiC/CE Nanocomposites

SiC nanofibers (100 mg) were placed in a flask (50 mL) and 20 mL of deionized water was added. The mixture was mixed on a magnetic stirrer (1000 rpm) for 2 min at 25 °C. With continuous stirring, 2 mL of HCl (diluted in water 1:1) was added and 2 mL of a saturated solution of CE (≈0.4 g·100 mL^−1^) in Schweizer’s reagent (synthesized in laboratory from Cu(OH)_2_ and ammonia) was added in small portions. If required, HCl was added to adjust the pH to ~7. The obtained suspension was centrifuged (0.5 min, 1000× *g*) and washed in deionized water. The resulting product was placed on a filter, washed with water three times, and then freeze dried for 12 h.

### 3.3. Synthesis of SiC/Ag/CE Nanocomposites

The nanocomposite was synthesized in two different reaction mediums. In one case, a solution of sodium dodecyl sulfate (SDS) was used (Sigma–Aldrich, Sigma–Aldrich Sp. z.o.o, Poznan, Poland) to form a SiC/Ag/CE/SDS composite. In the second case, Tween 20 (Chemsolve, WITKO Sp. z.o.o, Łódź, Poland) was used to obtain a SiC/Ag/CE/T nanocomposite. To the flask (50 mL), 100 mg of SiC nanofibers and 20 mL of an aqueous solution of SDS (1%) or Tween 20 (1%) were added. The mixture was stirred on a magnetic stirrer (1000 rpm) for 2 min at 25 °C. Then, with continuous stirring, 200 μL of solution of AgNO_3_ (0.5 M) and 100 μL of aqueous solution of hydrazine (80%, Avantor Performance Materials, Gliwice, Poland) were added. Hydrazine solution was added dropwise in portions. The mixture was left for ~5 min with continuous stirring, followed by the addition of 2 mL HCl (diluted in water 1:1) and then 2 mL of a saturated solution of CE in Schweizer’s reagent in small portions. If required, HCl was added again to adjust the pH to ~7–8 and CE solution addition was continued. The obtained nanocomposite suspension was centrifuged (0.5 min, 1000× *g*) and washed in deionized water. This was repeated at least five times to remove SDS or Tween 20. Next, the product was placed on a filter, washed with water three times, and then freeze dried for 12 h. The hydrazine was chosen as a reducing agent to avoid side products and impurities, which are introduced into the final nanomaterial when other compounds, such as borohydrides, organic aluminum hydrides or phosphites, are used.

### 3.4. Synthesis of AgNPs

AgNPs were synthesized to evaluate the influence of reaction conditions on nanoparticle size. 200 μL AgNO_3_ (0.5 M) was added to the 20 mL of SDS solution (1%) or Tween 20 solution (1%). Next, 100 μL hydrazine (80%) was added in small portions. The synthesis conditions were the same as those described above (magnetic stirrer, temperature), but the suspension did not contain SiC nanofibers. Then, HCl and ammonia solutions (25%, instead of a CE solution) were added in the same proportion with a pH control, as described above. Next, the obtained AgNPs (AgNP/SDS and AgNP/T) were washed and centrifuged twice (2 min, 15,000× *g*) and suspended in deionized water.

### 3.5. Composites Properties

The SiC nanofibers, nanocomposites, and AgNPs were examined using SEM without coating (Sigma VP, Carl Zeiss Microscopy GmbH, Oberkochen, Germany). To evaluate the distribution of AgNPs in the nanocomposites, energy-dispersive X-ray spectroscopy (Bruker, Berlin, Germany) mapping was conducted. X-ray fluorescence (XRF) analysis of the water suspension of SiC nanofibers and nanocomposites was used to determine the AgNP content of the nanocomposites. This analysis was conducted on an XRF spectrometer (Panalytical MiniPal 4, Almelo, The Netherlands) at 20 kV, 200 μA and with an Al-filter for 60 s. The aqueous suspension of AgNPs was used as a standard. XRD spectra of SiC nanofibers and nanocomposites were measured (Panalytical X’ Pert PRO MPD, Almelo, The Netherlands, Cu Kα radiation) for 2θ from 10° to 95°. The ultraviolet-visible (UV-VIS) spectra of AgNPs were obtained with a UV-VIS spectrophotometer (Genesys 10S UV-VIS, Thermo Fisher Scientific, Madison, WI, USA).

### 3.6. Microorganisms and Media

*Escherichia coli* (ATCC 8739) and *Bacillus cereus* (ATCC 11778) strains were obtained from our collection of isolated microorganisms (Geomicrobiology Laboratory, Faculty of Geology, University of Warsaw, Warsaw, Poland). The taxonomic affiliation was confirmed by sequencing analysis of the 16S rDNA gene. The bacteria were cultivated in liquid and solid tryptic soy broth (TSB, Sigma–Aldrich, Sigma–Aldrich Sp. z.o.o, Poznań, Poland) comprised of the following (g·L^−1^): casein peptone, 17; soya peptone, 3; glucose, 2.5; NaCl, 5; K_2_HPO_4_, 2.5 and agar (as solid medium), 20. The final pH was 7.3. The media were autoclaved at 121 °C for 15 min.

### 3.7. Measurement of CO_2_ in Cultures with Nanocomposites

Respirometry analyses of microbial cultures with different amounts of nanocomposites were implemented to estimate the minimum inhibitory concentration (MIC). A MicroOxymax (Columbus Instruments, Columbus, OH, USA) respirometer was used to measure the amount of CO_2_ formed by the microbial activity of *E. coli* and *B. cereus*. The CO_2_ sensor range was 0%–15% volume. The test was conducted as follows. Ten milliliters of sterile medium (TSB) and 0.2, 0.5, 1, and 2 mg·mL^−1^ of investigated materials were placed in a 100-mL sterile glass bottle (Simax, Alchem Sp. z.o.o, Warsaw, Poland). Then, 0.5 mL of bacteria suspension (approximately 10^8^ colony-forming units (cfu) mL^−1^ in 0.9% NaCl) was added and the bottle was connected to a respirometric system. The cultures were stirred (120 rpm) at 25 °C for 48 h. The amount of CO_2_ produced was measured automatically every 2 h. The tests were repeated twice. Control experiments were conducted under the same conditions without nanostructures.

### 3.8. Measurement of Dehydrogenases Activity

Dehydrogenases activities in cultures after treatment with SiC nanofibers and nanocomposites were determined in triplicate using triphenyltetrazolium chloride (TTC). TTC can be used by microorganisms as an electron acceptor, and the production of insoluble formazan from TTC reflects dehydrogenases activity [32]. Ten milliliters of TSB medium was mixed with 10 mg (ca. 0.5 MIC) of investigated nanomaterials in a 100-mL sterile glass bottle (Simax). Next, 1 mL of inoculum was added (approximately 10^8^ cfu/mL in 0.9% NaCl) and the obtained suspension was stirred for 6 h (120 rpm) at 25 °C. Then, 5 mL of the suspension was added to a test tube. Subsequently, 1 mL of 3% TTC and 50 mg of CaCO_3_ were added (CaCO_3_ was added to maintain a neutral pH). The test tube was sealed using parafilm and incubated for 1 h at 30 °C in darkness. After incubation, 0.5 mL of 37% formaldehyde was added and the suspension was filtered through a 0.45-μm filter (Merck-Millipore, Merck Sp. z.o.o, Warsaw, Poland). Formazan retained on the filter was extracted with 96% ethanol and the solution color was determined by spectrophotometry (Genesys UV-VIS, Thermo Fisher Scientific, Madison, WI, USA). Dehydrogenases activity was measured as the amount of TTC reduced to formazan per hour in relation to the protein content (μg·h^−1^·mg-protein^−1^). The control was a culture incubated without nanostructures. To assess whether the investigated materials can interfere with measurements of dehydrogenases activity, an abiotic control was conducted. This test was performed under the same conditions described above, using a sterile medium without bacteria.

### 3.9. Catalase Activity

Catalase activity was conducted in the same cultures described above with dehydrogenase activity according to Luck [33]. Approximately 100 μL of culture was added to the reaction volume of 3 mL containing PBS buffer (pH 7.0) and 10 mM H_2_O_2_. The rate of change of absorbance at 240 nm was recorded (dA·min^−1^). Catalase activity was calculated by using the molar extinction coefficient of 43.6 L^−1^·M^−1^·cm^−1^. The enzyme activity was expressed as μmol H_2_O_2_ consumed min^−1^·mg-protein^−1^. In additional experiments, the interference with SiC nanofibers and nanocomposites under the same conditions, but without bacteria, was evaluated.

### 3.10. Viability Test

The viability test was performed according to Szala and Borkowski [9]. Briefly, to analyze the loss of viability, solutions of propidium iodide (PI) (2 mg, 0.1 L^−1^, pH 7.4) and acridine orange (AO) (5 mg 0.1 L^−1^, pH 7.4) were prepared in phosphate buffer. Four milliliters of sterile saline solution (0.9% NaCl) and 5 mg of the investigated nanomaterials were added (ca. 0.5 MIC) to a 20-mL glass bottle. Subsequently, 1 mL of *E. coli* or *B. cereus* suspension (approximately 10^8^ cfu mL^−1^ in 0.9% NaCl) was added to the mixture and mixed for 120 min at 25 °C (200 rpm). Then, the suspension was mixed with sucrose (60%) to separate unadsorbed bacteria. After centrifugation (2 min, 2600× *g*), the residue and unadsorbed bacteria in the supernatant were stained as follows. An amount of 0.2 mL of the supernatant or residuum and 30 μL of PI were added to a test tube and left for 10 min in darkness. Later, 15 μL of AO was added (staining for 2 min) to the test tube. After staining, 10 μL of the suspension was placed in a microscopic glass slide and covered with a coverslip. Next, ten representative fluorescence images of cells adsorbed onto the nanocomposite aggregate surface were acquired using an epifluorescence microscope with a B-filter. Results from the microscopic analysis were expressed as a ratio of the number of cells stained with PI (red–orange) divided by the number of cells stained with PI plus cells stained with AO (green).

### 3.11. Protein Measurements

To analyze the number of bacteria without using the cultivation method, the correlation between bacterial protein content and optical density (at λ = 600 nm) was plotted for *E. coli* and *B. cereus* separately. Protein measurement was conducted according to the Lowry method with some modifications [34].

### 3.12. Statistical Analysis

The obtained data (viability test) were analyzed for significant mean differences using a one-way analysis of variance (ANOVA) at *p* < 0.05. Post hoc tests for pair-wise differences and the identification of homogeneous subgroups were conducted using Tukey’s honestly significant difference procedure. Homogenous subgroups are indicated by diagrams marked with the same lowercase letters. ANOVA was computed with Statistica 10 software (StatSoft. Inc., Tulsa, OK, USA).

## 4. Conclusions

SiC nanofibers can function as good carriers for silver nanoparticles. Such nanocomposites, together with CE as a stabilizer, may be materials with useful antibacterial properties. The tested nanocomposites showed antibacterial activity, probably because of the generation of oxidative stress in cells. Mechanical damage to membrane integrity appeared to be less important. Nanocomposites that contained Ag exhibited stronger bactericidal effects than the SiC nanofibers. The addition of CE reduced the effects of mechanical damage and can potentially increase the adsorption of negatively charged bacterial cells to the nanocomposite aggregate in an aqueous suspension. However, this hypothesis should be confirmed in future experiments. The obtained nanocomposites may have practical application in air- and water-purifying systems. Active filters based on SiC nanofibers and AgNPs may have strong bactericidal properties, and, thus, may not have the disadvantages of typical filters that are based on activated carbon.
